# Supplementary material for: Orthogonal inducible control of Cas13 circuits enables programmable RNA regulation in mammalian cells
Source: Nat Commun. 2024 Feb 21;15:1572. doi: 10.1038/s41467-024-45795-x (PMC10881482; doi:10.1038/s41467-024-45795-x)
Supplement: Supplementary file 2 — Reporting Summary [file 41467_2024_45795_MOESM2_ESM.pdf]

nature portfolio

Corresponding author(s): Wilson Wong

Last updated by author(s): Jan 11, 2024

Reporting Summary

Nature Portfolio wishes to improve the reproducibility of the work that we publish. This form provides structure for consistency and transparency in reporting. For further information on Nature Portfolio policies, see our Editorial Policies and the Editorial Policy Checklist.

Statistics

For all statistical analyses, confirm that the following items are present in the figure legend, table legend, main text, or Methods section.

n/a	Confirmed	
☐	☒	The exact sample size (*n*) for each experimental group/condition, given as a discrete number and unit of measurement
☐	☒	A statement on whether measurements were taken from distinct samples or whether the same sample was measured repeatedly
☐	☒	The statistical test(s) used AND whether they are one- or two-sided<br>*Only common tests should be described solely by name; describe more complex techniques in the Methods section.*
☒	☐	A description of all covariates tested
☒	☐	A description of any assumptions or corrections, such as tests of normality and adjustment for multiple comparisons
☐	☒	A full description of the statistical parameters including central tendency (e.g. means) or other basic estimates (e.g. regression coefficient) AND variation (e.g. standard deviation) or associated estimates of uncertainty (e.g. confidence intervals)
☐	☒	For null hypothesis testing, the test statistic (e.g. *F*, *t*, *r*) with confidence intervals, effect sizes, degrees of freedom and *P* value noted<br>*Give P values as exact values whenever suitable.*
☒	☐	For Bayesian analysis, information on the choice of priors and Markov chain Monte Carlo settings
☒	☐	For hierarchical and complex designs, identification of the appropriate level for tests and full reporting of outcomes
☒	☐	Estimates of effect sizes (e.g. Cohen's *d*, Pearson's *r*), indicating how they were calculated

Our web collection on statistics for biologists contains articles on many of the points above.

Software and code

Policy information about availability of computer code

Data collection	Attune NxT Software for flow cytometry data collection; Gen5 for fluorescence microscopy data collection, PerkinElmer's Living Image software for in vivo luminescence data collection
Data analysis	FlowJo_v10.8 for flow cytometry data processing; Microsoft Excel and GraphPad Prism for data analysis

For manuscripts utilizing custom algorithms or software that are central to the research but not yet described in published literature, software must be made available to editors and reviewers. We strongly encourage code deposition in a community repository (e.g. GitHub). See the Nature Portfolio guidelines for submitting code & software for further information.

Data

Policy information about availability of data

All manuscripts must include a data availability statement. This statement should provide the following information, where applicable:

- Accession codes, unique identifiers, or web links for publicly available datasets
- A description of any restrictions on data availability
- For clinical datasets or third party data, please ensure that the statement adheres to our policy

Plasmids are available upon requests Source data of main figures are provided with this paper. Raw .fcs files and data for supplementary figures are available upon request to the corresponding author.

Research involving human participants, their data, or biological material

Policy information about studies with human participants or human data. See also policy information about sex, gender (identity/presentation), and sexual orientation and race, ethnicity and racism.

Reporting on sex and gender	Donor identity disguised.
Reporting on race, ethnicity, or other socially relevant groupings	Donor identity disguised.
Population characteristics	Donor identity disguised.
Recruitment	Boston Children's Hospital Blood Donation Center
Ethics oversight	Boston University IBC protocol: 18-1537

Note that full information on the approval of the study protocol must also be provided in the manuscript.

Field-specific reporting

Please select the one below that is the best fit for your research. If you are not sure, read the appropriate sections before making your selection.

[x] Life sciences [ ] Behavioural & social sciences [ ] Ecological, evolutionary & environmental sciences

For a reference copy of the document with all sections, see nature.com/documents/nr-reporting-summary-flat.pdf

Life sciences study design

All studies must disclose on these points even when the disclosure is negative.

Sample size	For all in vitro assays, sample size was determined by the number of wells or independent replicates included in the data. All data represents a sample size of three or larger. For all in vivo data, sample size was determined by the number of mice (N=1 for each mouse). All data represents a sample size of three or larger. A sample size of n = 3 (three experimental repeats) was chosen because (1) three is the minimum number to estimate variance in sample statistics and (2) in order to ensure individual measurements were not artifacts. For Figure 5E and F, and Supplementary Fig. 16E, as the number of conditions in the experiment is so big that if including more than one replicates for each condition, we would not be able to generate data every 2 hours, thus, the resolution of the experiment would have been reduced. Instead, the same experiment was repeated 3 times, and a representative experiment is displayed. All experiment showed same trend as we interpreted in the manuscript.
Data exclusions	No data were excluded.
Replication	In vitro assays were performed at least 2 times with similar results. Live cell imaging for the dual-regulated circuit experiment were performed once with triplicates (Figure 5e, f, and Supplementary Figure 16e), but the same experiment was performed multiple times with a single well for each drug concentration, and the same trend in adaptation topology across concentration variations was observed. In vivo experiments (Figure 6b, c, and d) were performed in multiple batches with N=4--6 for each batch. Each batch includes at least one sample for each condition and the results were similar between each batch and combined to form the final data set. All experiments were performed with at least 3 replicates. For Figure 5E and F, and Supplementary Fig. 16E, as the number of conditions in the experiment is so big that if including more than one replicates for each condition, we would not be able to generate data every 2 hours, thus, the resolution of the experiment would have been reduced. Instead, the same experiment was repeated 3 times, and a representative experiment is displayed. All experiment showed same trend as we interpreted in the manuscript.
Randomization	For in vitro experiment, samples are prepared all at once and run plates by plates with no special allocation of samples within or outside of a testing group. For in vivo experiments, after hydrodynamic tail vein injection for the delivery of plasmid DNA, mice were randomly selected for inducer or vehicle control administration. For in vitro experiments, within replicates transected or transduced with the same DNA mix, randomly selected triplicates are supplemented with inducers or vehicle control. : Experiments were performed on immortalized cell lines which can reasonably be assumed to be identical when split into multiple wells for trasnfection/infection. Thus, cells being input into different experimental conditions are effectively randomized without explicitly controlling covariates.
Blinding	For all experiments, Success metrics for our controller design vs the unregulated system were pre-defined (Relative MFI, leakiness, and inducibility).During in vivo luminescence imaging, the investigators are blinded to treatment mouse groups.

Reporting for specific materials, systems and methods

We require information from authors about some types of materials, experimental systems and methods used in many studies. Here, indicate whether each material, system or method listed is relevant to your study. If you are not sure if a list item applies to your research, read the appropriate section before selecting a response.

Materials & experimental systems

n/a	Involved in the study
☐	☒ Antibodies
☐	☐ Eukaryotic cell lines
☒	☐ Palaeontology and archaeology
☐	☒ Animals and other organisms
☒	☐ Clinical data
☒	☐ Dual use research of concern
☒	☐ Plants

Methods

n/a	Involved in the study
☒	☐ ChIP-seq
☐	☒ Flow cytometry
☒	☐ MRI-based neuroimaging

Antibodies

Antibodies used	HLA-ABA FITC conjugated antibody BD Bioscience 555552<br>HLA-A2 PE conjugated antibody BD Biosciences 560964<br>CD46 Alexa Fluor 594-conjugated Antibody R&D systems FAB2005T<br>ImmunoCult Human CD3/CD28 T Cell Activator STEMCELL 10971
Validation	All antibodies were used based on the validation statements on the manufacturer's website, including the specie and applications.

Eukaryotic cell lines

Policy information about cell lines and Sex and Gender in Research

Cell line source(s)	Lenti-X 293T (Takara Biosciences)<br>HEK293FT N/A<br>Jurkat clone E6-1 ATCC TIB-152<br>Neuro-2a ATCC CCL-131<br>U87MG N/A
Authentication	Cells were purchased and used directly from the source with no additional authentication.
Mycoplasma contamination	All cell lines tested negative for mycoplasma.
Commonly misidentified lines<br>(See ICLAC register)	No commonly misidentified cells were used in this study.

Animals and other research organisms

Policy information about studies involving animals; ARRIVE guidelines recommended for reporting animal research, and Sex and Gender in Research

Laboratory animals	Balb/c (female 5-6 weeks) Jackson Laboratory 005557 12 hours light /dark cycles; temp 68-79F, humidity 30-70%
Wild animals	The study did not involve wild animals
Reporting on sex	All mice involved in this study were female.
Field-collected samples	The study did not involve field collected samples
Ethics oversight	Our studies were overseen by the IACUC at Boston University

Note that full information on the approval of the study protocol must also be provided in the manuscript.

Plants

Seed stocks	N/A
Novel plant genotypes	N/A
Authentication	N/A

Flow Cytometry

Plots

Confirm that:

☒ The axis labels state the marker and fluorochrome used (e.g. CD4-FITC).

☒ The axis scales are clearly visible. Include numbers along axes only for bottom left plot of group (a 'group' is an analysis of identical markers).

☒ All plots are contour plots with outliers or pseudocolor plots.

☒ A numerical value for number of cells or percentage (with statistics) is provided.

Methodology

Sample preparation	Adherent cells were trypsinized and neuralized and suspended before flowcytometry. When surface protein expression were to be detected, cells were washed with FACS buffer (PBS without calcium + 0.1% NaN3 + 1% BSA + 2mM EDTA), stained with antibody, and washed with FACS buffer at least twice before flow cytometry.
Instrument	ThermoFisher Scientific Attune NxT Flow Cytometer
Software	FlowJo
Cell population abundance	All reported samples include at least 10,000 events.
Gating strategy	A polygonal FSC-A/SSC-A gate was used to remove debris and dead cells from the mammalian cell populations. Gates for successfully transfected or transduced populations were drawn based on the marker expression in non-transfected or no-transduced (negative) controls.

☒ Tick this box to confirm that a figure exemplifying the gating strategy is provided in the Supplementary Information.
